# Long-term efficacy of sclerosing foam combined with endovenous laser treatment for varicose veins of the lower extremities

**DOI:** 10.3389/fsurg.2025.1639750

**Published:** 2026-01-14

**Authors:** Guili Wang, Donglin Lu, Ding Wu, Xiaoli Wang, Yi Rong, Xiwen Liu, Zhaoxuan Liu

**Affiliations:** Department of Vascular Surgery, Central Hospital Affiliated to Shandong First Medical University, Jinan, China

**Keywords:** symptoms, sclerosing foam, vascular surgery, lower extremity varicose veins, long-term effect

## Abstract

**Background:**

This investigation aimed to assess the long-term effectiveness and symptomatic manifestations of the combined therapy of sclerosing foam and endovenous laser ablation (ELA) for treating lower-extremity varicose veins.

**Materials and methods:**

In this study, we examined 2,118 patients (2,324 limbs) diagnosed with varicose veins in one or both lower extremities. These patients were treated at our center between January 2019 and December 2021. All individuals underwent the combined treatment of sclerosing foam and ELA. We closely monitored the occlusion status of the great saphenous vein (GSV) trunk and its tributaries, along with the postoperative therapeutic outcomes and symptomatic presentations. The average follow-up duration was 41.3 months, and data were collected via outpatient appointments and telephone follow-up inquiries.

**Results:**

The success rate of the treatment procedure was 100%. Based on the outcomes of continuous follow-up spanning from 1 to 3 years after the surgery, among the 2,324 legs, 16 legs still required repeated foam sclerotherapy for varicose veins, 2 legs exhibited venous edema, and 11 legs showed skin pigmentation. At one week and one year after the procedure, incomplete closure of the GSV trunk (characterized by patency, blood flow, and reflux) was observed in 1.8% (38 limbs) and 1.1% (23 limbs), respectively. Nineteen patients with local recurrence of varicose veins decided not to undergo further treatment.

**Conclusion:**

The long-term follow-up data demonstrated that the combination of sclerosing foam and endovenous laser was highly efficacious in treating varicose veins. The main postoperative symptoms included the requirement for additional sclerosing foam injections, skin pigmentation, pain and tissue induration.

## Introduction

Varicose veins, a common vascular disorder characterized by venous insufficiency and reflux, significantly affect the quality of life of millions of people, especially in the lower extremities. This condition is associated with a high level of morbidity, including chronic pain, swelling, skin pigmentation changes, and the potential development of severe complications such as venous ulcers and thromboembolic events (1, 2). Over the past two decades, minimally invasive treatments have emerged as the preferred option for managing varicose veins, offering advantages over traditional surgical methods in terms of efficacy, safety, and patient satisfaction (3, 4).

Among the various minimally invasive techniques, endovenous laser ablation (ELA) and sclerosing foam therapy have been widely accepted. ELA uses thermal energy to cause thermal injury and subsequent closure of the great saphenous vein (GSV), while sclerosing foam, a form of chemical ablation, causes endothelial damage and fibrosis of the venous wall (5, 6). Both techniques have shown high success rates in achieving venous occlusion and improving clinical symptoms. However, individual limitations, such as incomplete closure of venous tributaries and recurrence rates, have led to the exploration of combined therapies to improve treatment outcomes (7, 8).

The combination of sclerosing foam and ELA has been proposed as a synergistic approach to overcome the limitations of monotherapy. By targeting both the main trunk of the GSV and its tributaries, this combined modality aims to achieve more complete venous closure, reduce recurrence rates, and minimize postoperative complications. Recent studies have reported favorable short-term results with this approach, including high procedural success rates and significant improvement in clinical symptoms (9, 10). However, long-term outcomes, particularly regarding the persistence of symptoms and the need for additional interventions, remain less well-documented.

Despite the increasing evidence supporting the use of combined therapies, there are few studies focusing on the long-term efficacy and symptom profiles of sclerosing foam combined with ELA. Furthermore, the identification of predictors of recurrence and the optimization of treatment protocols still need further investigation. This study aims to fill these gaps by providing a comprehensive evaluation of the long-term outcomes of sclerosing foam combined with ELA in the treatment of lower-extremity varicose veins, with a particular focus on procedural success, symptom resolution, and the need for additional interventions.

## Method

### Study design

This retrospective single-center study strictly adhered to the principles of the Declaration of Helsinki, ensuring that there were no privacy risks to the enrolled patients. As a retrospective clinical study, the Ethics Committee of the Affiliated Central Hospital of Shandong First Medical University waived the need for informed consent.

We conducted a retrospective review of 2,118 patients (2,324 limbs) diagnosed with varicose veins in one or both lower extremities, who received treatment at our center from January 2019 to December 2021. All the enrolled patients had Clinical Etiological Anatomical Pathophysiological (CEAP) C2–C6 varicose veins, characterized by tortuous lesions in the great saphenous vein and its branches. Patients with simple small saphenous vein varicose veins and deep vein thrombosis were excluded from the study. Before surgery, patients were admitted to the hospital for comprehensive preoperative assessments, including electrocardiography, blood routine tests, and lower-limb venous anterograde angiography. The ultrasound findings of the enrolled patients were as follows: during quiet breathing, an internal diameter of the great saphenous vein exceeding 4 mm was generally considered an abnormal dilation. After taking a deep breath, holding it, and then making a forced exhalation (Valsalva maneuver), blood reflux was observed, and the reflux duration was longer than 1 s.

### Materials preparation

The materials used in this study included lauromacrogol sclerosing foam (H20080445; Shanxi Tianyu Pharmaceutical, China), an LFK-SLT30 semiconductor laser therapy instrument, low-temperature optical fiber (Laser parameters: 1,470 nm wavelength, radial-emitting fiber, power set at 12–18 W, linear endovenous energy density: 80–100 J/cm), a 5F venipuncture cannula, guidewire, catheter, tumescent anesthesia solution, and Doppler ultrasound.

### Surgical methods

A total of 2,118 enrolled patients were diagnosed through duplex ultrasound imaging of the lower-limb veins. This imaging modality was used to precisely identify the presence, location, and extent of varicose veins, as well as to evaluate the patency and function of the venous system. Prior to the surgical procedure, all patients received tumescent anesthesia. Before the administration of anesthesia, varicose veins were carefully marked on the skin using a surgical marking pen. This step was crucial for accurately identifying the target veins during the operation. The Seldinger technique was then employed to puncture the GSV at the knee level. Under real-time ultrasonographic guidance, the laser fiber tip was positioned at least 2 cm distal to the saphenofemoral junction. This distance was strictly adhered to in order to avoid potential damage to the saphenofemoral junction and to ensure the safety and effectiveness of the subsequent treatment.

Subsequently, under ultrasound guidance, tumescent anesthesia was injected around the GSV in the groin area. The purpose of this anesthesia was not only to provide local pain relief but also to compress the GSV, facilitating better visualization and subsequent treatment. Multi-site puncture treatment was carried out on the main stem of the GSV, the diseased small saphenous vein, and all clinically visible tributaries. After the punctures were made, a foam sclerosant (product code: H20080445; manufactured by Shaanxi Tianyu Pharmaceutical) was injected. The sclerosing foam was prepared using the Tessari method, with a ratio of 1:3 sclerosant to air, This specific ratio was chosen to optimize the foam properties for sclerosing the target veins. The prepared foam was then used to repeatedly flush the target vessels to ensure uniform contact of the sclerosant with the vessel walls. The varicose veins were re-marked as necessary, and a scalp needle was used to puncture the identified sites. This allowed for more precise delivery of any additional treatments if required.

Next, laser thermal ablation was performed on the GSV trunk that had been pre-treated with the foam sclerosing agent. The laser instrument was set to an output power of 12–18 W and a total laser energy level of 2,800–3,600 J. The laser guidewire was advanced at a speed of 0.5–1 cm/s. The advancement was continued until complete closure of the GSV trunk above the knee joint was achieved. In cases where vessel twisting or tortuosity hindered the smooth passage of the optical fiber, a segmented puncture and closure approach was adopted. In this approach, the affected vessel segment was divided into smaller sections, and each section was punctured and treated separately until the entire segment was successfully closed.

Immediately after the treatment was completed, patients were instructed to wear medical elastic stockings. These stockings were used to provide external compression, promote venous return, and reduce the risk of postoperative hematoma and edema.

Patients who were asymptomatic after the surgery were discharged on the following day. After exclusion of relevant contraindications, all patients were required to wear the Class II medical elastic stockings continuously for at least one week, and received unified anticoagulant therapy for one week after surgery (rivaroxaban 10 mg daily). Additionally, they received thorough rehabilitation education, which included instructions on wound care, activity restrictions, and the importance of regular follow-up visits to monitor the recovery process.

### Observation indices and statistical analysis

Postoperative follow-up point at hospital stays of the patients, the cure of Varicose veins of the lower extremities (VVLEs), and surgical symptoms (such as postoperative subjective pain, saphenous nerve injury, deep vein thrombosis, venous edema, varicose veins, and induration). To measure the efficacy of surgery, within 12 months following the initial procedure, each patient received outpatient follow-up visits (1–12 times). We recommend that patients visit the outpatient department for follow-up once a month within the first six months after surgery. The decision to extend the follow-up period will be based on the outcomes of the sixth-month follow-up. Telephone follow-up was conducted at 1, 3, 6, 12, 24 and 36 months after surgery. During outpatient follow-up, medication and/or ambulatory rehabilitation including analgesic, anti-inflammatory, and detumescence was provided to cope with postoperative symptoms, such as persistent pain, induration, and phlebitis (11). Based on the specific postoperative recovery status of patients, it should be determined whether it is necessary to reduce the duration of wearing elastic stockings or switch to low pressure short tube elastic stockings to alleviate lower limb swelling. For patients with lower extremity varicose veins and ulcers, during the operation, a sclerosing agent should be injected into the blood vessels surrounding the ulcer to reduce blood flow. After the operation, the dressing should be changed every two to three days. The statistical analysis of categorical variables used chi-square tests to compare the measurement results of categorical data between groups. The *P*-value less than 0.05 was considered statistically significant. A summary, calculation, and comparison of all data were performed in SPSS Statistics (IBM Corp, USA). All follow-up work and measurements were independently performed by two physicians.

## Results

The surgery success rate was 100%. Refers to technical success immediate postoperative ultrasound examination showed the trunk and clinically visible tributaries of the GSV had closed well. From January 2019 to December 2021, 2,118 patients (2,324 legs) with VVLEs (1,169 males, 1,155 females; mean age: 59.3 ± 11.2 years) were identified. All patients presented with CEAP classification C2–C6 varicose veins: 206 with bilateral VVLEs and 1,912 with unilateral VVLEs. Limbs were categorized as C2–C4 (2,237 limbs), C5–C6 (87 limbs). Tables 1, 2 provides detailed baseline information on all 2,118 patients. During the study period, a total of 2,324 legs received sclerosing foam combined with laser treatment for VVLEs, and the 2,098 legs included in the study, 226 were lost to follow-up (Table 3). The mean follow-up time was 41.3 ± 5.2 (ranging from 33 to 50) months, and the follow-up rate was 90.3% (2,098/2,324).

**Table 1:** Baseline characteristics of 2,118 screened patients.

Dimension	Max	Min	Mean	SD	Median
Age	91	19	59.3	11.2	60
Hospital stays	35	1	3.6	3.1	2
Months of follow-up	50	33	41.3	5.2	41

SD, standard deviation.

**Table 2:** Distribution of main admission diagnoses among 2,324 affected limbs with lower limb varicose veins.

Primary diagnosis	Count	Proportion
Post-varicose vein surgery	36	1.5%
Iliac vein compression syndrome	18	0.8%
Lower limb varicose veins	2,071	89.1%
Lower limb varicose veins with phlebitis	23	1.0%
Lower limb varicose veins with ulcers and inflammation	87	3.7%
Lower limb varicose veins with phlebitis and superficial vein thrombosis	89	3.8%
Total	2,324	100%

**Table 3:** The efficacy of the treatments.

Dimension	*n*, (%)
All leg lesions	2,324
Follow up	2,098 (90.3)
Loss of follow-up	226 (9.7)
Surgery success	2,324 (100)
Symptoms at 1 week–12 months	379 (18.1)
Symptoms after more than 12 months	47 (2.2)
Blood-ﬂow signal at 1 week	38 (1.8)
Blood-ﬂow signal at 1 week–12 months	23 (1.1)

Early postoperative Doppler ultrasound scans were performed for all included cohort patients, 1 week after the procedure. These studies revealed that incomplete closure (Defined by duplex ultrasound as GSV trunk patency with detectable antegrade/retrograde flow, reflux duration >0.5 s, and vein diameter >3 mm at follow-up.) of the GSV trunk included 38 (1.8%) blood-ﬂow signal at 1 week and 23 limbs (1.1%) blood-ﬂow signal at 1 week–12 months, respectively. At 1 year–3 years following surgery, 11, 8, and 2 of all 2,098 legs still with skin pigmentation, induration, and venous edema, respectively. A total of 87 patients suffering from lower extremity varicose veins complicated by ulcers and inflammation were enrolled in this study. The follow-up outcomes within 1 year post operation indicated that 12 patients still had lower extremity venous ulcers. Moreover, the follow up results within 3 years after the operation revealed that only 2 cases remained with such ulcers. The Venous Clinical Severity Score is a standardized tool recommended by the International Union of Phlebology for quantitatively assessing the severity of chronic venous disease. We monitor treatment efficacy by scoring the clinical symptoms and signs of postoperative patients. A small number of patients experienced 2–3 symptoms. The major postoperative symptoms are shown in Tables 4, 5. The number of lower limbs with surgery related symptoms 1 year after surgery and 2–3 years after surgery were 379 and 47, respectively; It can be seen that the postoperative symptoms gradually improve over time, but some patients may have surgery related symptoms that persist for a longer period of time. No clinical episodes of pulmonary embolism or other cardiovascular symptoms were observed. There was a difference between the mid and long-incidence of symptoms (Table 6). The incidence of skin pigmentation (3.7% in mid vs. 0.5% in long-follow-up, *P* < 0.01) and induration (2.5% in the mid vs. 0.4% in long-follow-up, *P* < 0.01) was statistically significant between the treatment groups. From Table 6, it can be seen that there are statistically significant differences in the mid-term and long-term follow-up results of all surgical related symptoms. Within 12 months after surgery, 122 limbs received a repeat injection of sclerosing foam, and 11 patients were re-admitted for the same surgical treatment during follow-up. During 12–36 months after surgery, 16 limbs received a repeat injection of sclerosing foam.

**Table 4:** Venous clinical severity score at 1 week–12 months following surgery.

Item	Mild (1 point)	Moderate (2 points)	Severe (3 points)	All
Pain	45	16	11	72
Varicose veins	33	18	1	52
Venous edema	15	9	4	28
Skin pigmentation	38	33	7	78
Inflammation	15	8	1	24
Induration	30	19	3	52
Active ulcers	10	2	0	12
Compression therapy	44	17	0	61

**Table 5:** Venous clinical severity score at 1 year–3 years following surgery.

Item	Mild (1 point)	Moderate (2 points)	Severe (3 points)	All
Pain	2	2	0	4
Varicose veins	9	7	0	16
Venous edema	1	1	0	2
Skin pigmentation	8	3	0	11
Inflammation	0	1	0	1
Induration	7	1	0	8
Active ulcers	1	1	0	2
Compression therapy	2	1	0	3

**Table 6:** Comparison of venous clinical severity scores between short-term and long-term follow-up after surgery.

Dimension (1–3 points)	1 week–12 months (*n*, %)	12–36 months (*n*, %)	*P* value
Pain	72 (3.4)	4 (0.2)	<0.01
Varicose veins	52 (2.5)	16 (0.8)	<0.01
Venous edema	28 (1.3)	2 (0.1)	<0.01
Skin pigmentation	78 (3.7)	11 (0.5)	<0.01
Inflammation	24 (1.1)	1 (0.05)	<0.01
Induration	52 (2.5)	8 (0.4)	<0.01
Active ulcers	12 (0.6)	2 (0.1)	<0.01
Compression therapy	61 (2.9)	3 (0.14)	<0.01
All	379	47	<0.01

## Discussion

The current study evaluates the long-term outcomes of sclerosing foam combined with laser therapy for the treatment of VVLEs in a large cohort of patients. The results demonstrate a 100% surgical success rate, with immediate postoperative ultrasound examinations confirming the closure of the GSV trunk and its tributaries. The study followed 2,098 limbs (2,118 patients) over a mean period of 41.3 months, with a follow-up rate of 90.3%. The findings reveal that most postoperative symptoms, such as pain, venous edema, skin pigmentation, and induration, significantly improved over time, although a small proportion of patients experienced persistent symptoms. The immediate success of the procedure, as evidenced by the complete closure of the GSV trunk and tributaries, aligns with previous studies that have highlighted the efficacy of sclerosing foam and laser therapy in achieving venous closure (12, 13). These findings are consistent with the mechanism of action of sclerosing foam, which induces endothelial damage and subsequent fibrosis, leading to venous occlusion (14). The absence of major complications, such as pulmonary embolism or cardiovascular events, underscores the safety of this minimally invasive approach compared to traditional surgical interventions (15). The study focuses on evaluating the long-term effectiveness of the hybrid therapy itself rather than comparing it to monotherapies (e.g., laser alone or foam alone).

**Figure 1:**
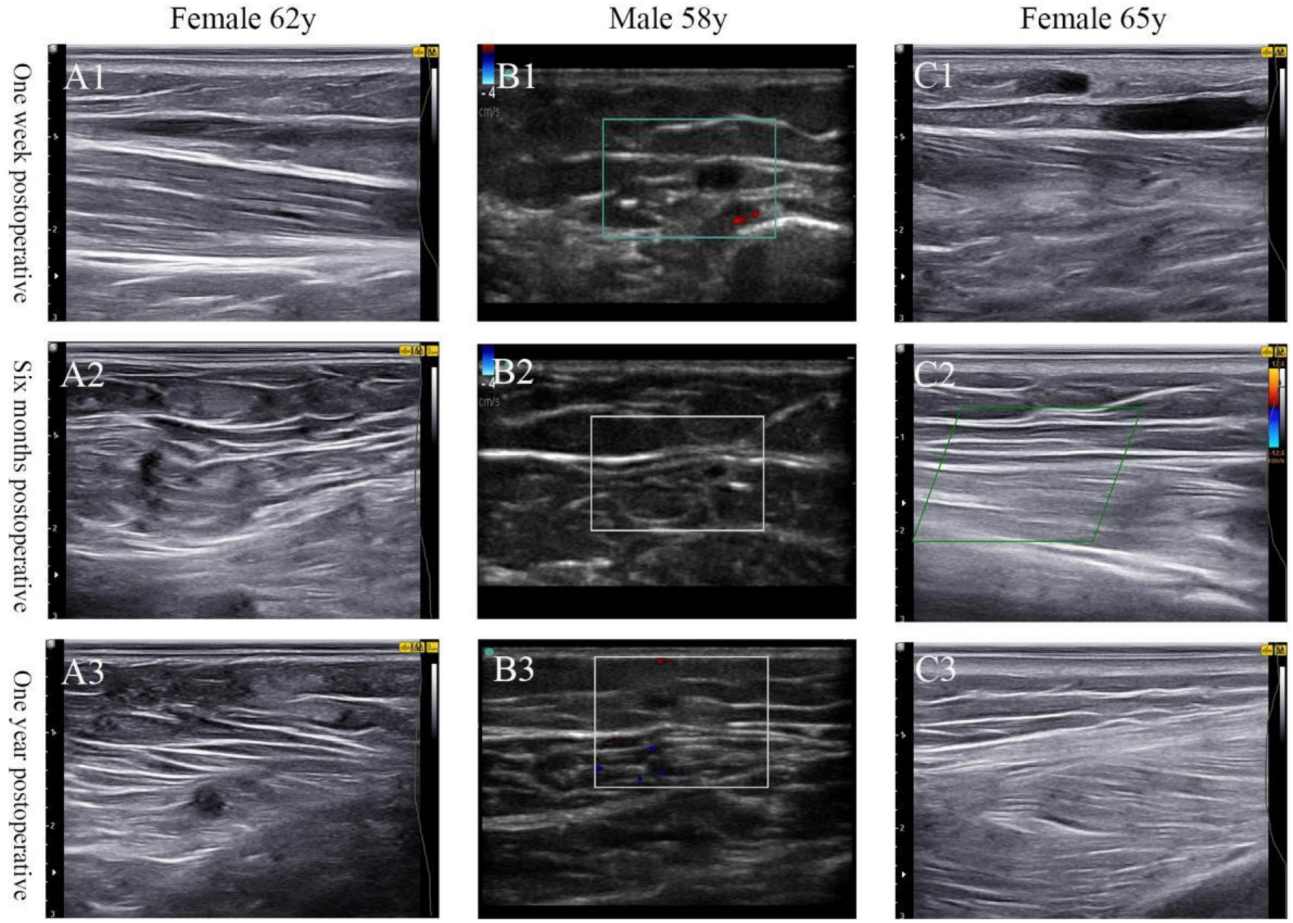
Follow-up ultrasound results of three patient post-surgery. **(A1,B1,C1)** One week postoperative, **(A2,B2,C2)** six months postoperative, **(A3,B3,C3)** one year postoperative.

The study highlights a significant reduction in postoperative symptoms over time. At 1 week to 12 months post-surgery, 379 limbs (18.1%) exhibited symptoms such as pain, venous edema, and skin pigmentation. However, by 1–3 years post-surgery, only 47 limbs (2.2%) reported persistent symptoms. This gradual improvement in symptoms is consistent with the natural healing process following venous closure, as previously reported in the literature (16, 17). The persistence of symptoms in a small subset of patients may be attributed to residual venous reflux or incomplete closure of smaller perforating veins, which are less detectable on routine ultrasound examinations (18, 19). The statistically significant differences in the incidence of symptoms between the mid-term (1 week–12 months) and long-term (12–36 months) follow-up periods (Table 6) suggest that the majority of patients experience a gradual resolution of symptoms over time. The ultrasonic follow-up results of three patients at 1 week after surgery are shown in Figures 1A1,B1,C1 while the same patients at 6 months after surgery are shown in Figures 1A2,B2,C2, and the ultrasound follow-up results one year after surgery showed that the main trunk of the GSV was well closed, as shown in Figures 1A3,B3,C3. The study of Jiang wenhong et al., shows that at 1 month, the occlusion rates of GSV were 98.35% for radiofrequency ablation and 98.04% for laser ablation, whereas at 1 year, the rates were 93.13% for radiofrequency ablation and 94.18% for laser ablation (20).

The need for repeat sclerosing foam injections in 122 limbs (5.3%) and re-admission for surgical treatment in 11 patients (0.5%) highlights the importance of individualized treatment plans and close monitoring in patients with VVLEs. Repeat treatments are often required in cases of incomplete venous closure or recurrent venous reflux, particularly in patients with advanced CEAP classifications (C5–C6) (21). The low rate of repeat treatments in this study may be attributed to the high success rate of the initial procedure, as well as the comprehensive preoperative assessment, which likely minimized the risk of incomplete closure. The findings of this study are consistent with those of several recent studies evaluating the outcomes of sclerosing foam and laser therapy for VVLEs. For instance, a study by Venermo et al. reported a 100% success rate for endovenous laser ablation and ultrasound-guided foam sclerotherapy in patients with varicose veins, with a significant reduction in symptoms over a 1-year follow-up period (22). Similarly, the study by Victoria et al. demonstrated that laser therapy combined with sclerosing foam was effective in achieving long-term venous closure and improving patient outcomes (23). However, some studies have reported higher rates of persistent symptoms, particularly in patients with advanced disease or comorbidities (24, 25). The lower rate of persistent symptoms in this study may be attributed to the inclusion of a larger proportion of patients with less severe disease (C2–C4) and the use of a combined sclerosing foam and laser therapy approach, which may enhance the efficacy of treatment.

While the study provides valuable insights into the long-term outcomes of sclerosing foam and laser therapy for VVLEs, there are some limitations that warrant consideration. First, the study relies on patient-reported symptoms and Doppler ultrasound findings, which may underestimate the true incidence of venous reflux or incomplete closure. Second, the study does not include a control group or comparison with alternative treatment modalities, limiting the ability to draw definitive conclusions about the relative efficacy of this approach. Third, We note that selection bias may arise from non-random patient enrollment (e.g., patients with more severe varicose veins may have been selected for hybrid therapy at our center). We also acknowledge that unmeasured confounding factors (e.g., operator experience, patient adherence to follow-up) could influence outcomes. Future studies should aim to address these limitations by incorporating objective measures of venous function, such as duplex ultrasound or venous pressure measurements, and by comparing the outcomes of sclerosing foam and laser therapy with other treatments, such as endovenous thermal ablation.

## Conclusions

In conclusion, this study demonstrates that sclerosing foam combined with laser therapy is a safe and effective treatment for VVLEs, characterized by a high surgical success rate and sustained, significant improvement in symptoms over time. Moreover, the findings underscore the importance of long-term follow-up and individualized treatment planning in optimizing outcomes for patients with varicose veins. While our data lend support to the hybrid approach as a promising therapeutic option in clinical practice, randomized controlled trials are needed to confirm its superiority.

## Data Availability

The raw data supporting the conclusions of this article will be made available by the authors, without undue reservation.
